# Seventh Conference of the Association of Hospital Superintendents

**Published:** 1905-10-21

**Authors:** 


### SEVENTH CONFERENCE OF THE ASSOCIATION OF HOSPITAL SUPERINTENDENTS.

### AMERICAN VIEWS AND OPINIONS.

At the opening session of the Seventh Conference of Hospital Superintendents held at Boston in the Hotel Vendome, September 26 to 29, Dr. George H. M. Rowe, of the Boston City Hospital, President of the Convention, presided. The Conference was opened by prayer by the Rev. Edward Everett Hale, D.D. Dr. David W. Cheever delivered an address of welcome, to which Dr. Henry M. Hurd, of the Johns Hopkins Hospital, Baltimore, responded. Dr. George H. M. Rowe gave an address in which he briefly reviewed the history of the association, whose principle was co-operative, democratic, and beneficial.

Sir Henry C. Burdett, K.C.B., made an appeal for the better financial management of hospitals. Sir Henry said in brief : "The control of hospitals by trustees and medical superintendents should be complete in everything but the treatment of disease, and each member of the medical staff should be paid. The need of the moment was to awaken the medical men to think of, to look at, and to consider the great cost of treatment; then they will gladly conform to the economies suggested by the trustees and the superintendent. The poor must be the first consideration, all the others should pay adequately. The accounts of each department should be kept separately, so that the working of the hospital can be seen, and the donors may know definitely to what use the money they have so generously given has been put.

### Afternoon Session.

*The Construction and Arrangement of Hospitals.*

Mr. Edmund M. Wheelright, architect, of Boston, spoke on "Hospital Operating-rooms and their Accessories." "The Standardising of Hospital Construction and Equipment" was the subject of a paper by Mr. Bertrand E. Taylor, architect, of Boston.

Mr. Wheelwright maintained that the hospital superintendent and architect should work together for one common aim, namely, the future usefulness of the building they are planning for their hospital. As for material, marble is preferable to cement because it does not need to be renewed; the walls should be of hard plaster; all woodwork excluded. The hospital demands no purely decorative system, and everything should have the virtue of cleanliness.

Mr. Taylor said that the problem of hospital building is primarily one of finances, which the great increase of cost of material tends to increase. The financial status of the hospital has not been given the attention it deserves, for numerous reasons.

*First.*—Competition in that line has been lacking.

*Second.*—Few hospitals are built all at once.

*Third.*—Hospital trustees are seldom of sufficient experience.

*Fourth.*—In large hospitals the superintendent is usually capable, but in small ones he is not usually a man of varied or practical experience.

*Fifth.*—The architect is not efficient.

Hospitals, like Topsy, are not born but grow, and as there is great difference of opinion in their construction it is almost impossible to fix uniform standards. There is an evolution in favour of small wards and single rooms, also the hospitals should be more like sanatoria in giving light and fresh air to patients. The hospital should have unit furniture and accessories as a precaution against dust.

In the discussion which followed these speeches, it was generally agreed that, owing to the different aims of the hospitals, as well as to the varying sums that could be expended on them, the standardising of hospitals was impossible. Dr. Renwick R. Ross, of the Buffalo General Hospital, of Buffalo, New York; Dr. Henry M. Hurd, of Baltimore; Sir Henry Burdett, of England; Dr. C. Irving Fisher, of New York; and Dr. S. F. Armstrong, of New York, took part in the discussion. The pavilion plan of hospital was generally favoured, also the provision of balconies, the floor of the balconies to be on a level with the floor of the ward, so that patients could be easily rolled out. The various questions of large and small, open and closed wards; heating, ventilating, and arrangement were freely discussed, and there were as many different ideas on those subjects as there were people to express them. Sir Henry Burdett gave the essentials of a hospital as absolute simplicity and cleanliness. His advice was to get back to nature and get real efficiency on simple and cheap lines. The necessity for closets for the patients' clothes, isolated rooms for very noisy or very sick patients, the advantage of large wards in the better care of the patients and better supervision of nurses, was demonstrated in a convincing talk by Miss Maud Banfield, of the Philadelphia Polyclinic. Dr. John M. Peters, of the Rhode Island Hospital, Providence, stated as his opinion, that no board of hospital administration was complete without a woman of continuous practical experience as a member of that board.

### Hospital Administration.

The Wednesday morning session was opened by a paper on "Hospital Administration," delivered by Mr. George P. Ludlam, chairman of the session. "To what extent has the increased Cost in Hospital Construction and Equipment been justified?" was the subject treated by Dr. S. S. Goldwater and Dr. Thomas Howell.

Dr. Moses Collins talked on "An aid in the perfecting of Hospital Management." He maintained that expensive hospitals are not necessary to good results, since contact, not surroundings, is the basis of all good surgery. Simple surroundings would result in economy of supplies by surgeons.

The discussion of these papers was led by Dr. Herbert B. Howard and Dr. William O. Mann. Dr. Howard proved conclusively the advantage of a convalescent home. Under the head of deficits he said that the large ones were due to the fact that the superintendent had no definite opposing wall of conditions which could say "Thus far shalt thou go and no farther." That a medical school in connection with a hospital is expensive, he did not deny. The superintendent should see that the students do not waste material, since such methods destroy the reputation of the hospital and give the students careless habits. He did not decry expensive buildings, if the building committee has the money to spend; but that money must be spent to attain perfection and not elegance. Certain kinds of experimentation are permissible; for instance, anything that has to do with the operating table and its equipment, for in that line the hospital has its own problems to solve. But as for engineering and electrical experiments, anything in that direction should be left to mechanics and engineers.

Sir Henry Burdett advocated the convalescent home or suburban branch hospital strongly. He said he had found, as he presumed had been the case with all his colleagues, that the atmosphere of a hospital ward was not good for patients when they are recovering; but that these same patients showed a rapid increase of vigour when sent to the country. There is also in favour of the Home, the condemnation of science against treating surgical cases in crowded districts. The time will come when the hospital system will be outside the town where people reside. He suggested that the deficit trouble be met by an organisation in the United States similar to that which exists in England under the name of "The League of Mercy." The keynote of the League is that the only true and unselfish way of giving is to give of one's self in personal service to the sick. The members of the League with health, wealth, and love of humanity, work in the days of health for those who are less fortunate than themselves.

At the close of the Session, Sir Henry Burdett was unanimously elected an honorary member of the Association.

Families and Tuberculosis.

The meeting on Wednesday night was held at the Boston Medical Library, in the Fenway. Dr. Henry M. Hurd read a paper on "John Howard's Observations on Hospitals, 1773-1790," in which he showed that the medical profession was even at that early date well acquainted with the contagiousness of tuberculosis. In the discussion which followed, Sir Henry Burdett delivered a protest against the fear of consumption being carried to such an extent as to result in inhuman acts of cruelty to those afflicted with this disease. Sis Henry said in brief :—"There is no such contagiousness about tuberculosis as should render it necessary for an exhibition of any of the contemptible panic which sometimes possesses families. For we know perfectly well that with ordinary precautions, and with the intelligent co-operation of the patient, steps may be taken that will render the disease, for all purposes of domestic treatment, reasonably safe. It ought, therefore, to be possible for a family to keep its dear ones near to it, and take care of them, when afflicted with this disease; and not to treat them as objects of danger to be shunned and avoided. While I advocate and support the fullest measures of prevention and disinfection, I also urge that we must be rational, and that in considering phthisis as an infectious disease, we are not to teach that it must be isolated on a hill, as near the sky as possible. I believe that many poor people have already been fatally injured by their friends because of this panic fear of consumption."

In supporting the view just expressed, Dr. Hurd said :—"I have no doubt that the patient who is instructed as to the danger to others, who is fully aware that people sympathise with him and are not afraid of him, will be less a source of danger than the neglected consumptive who is not thus instructed, who has not the sympathy of his friends, and who feels that he is an object of suspicion. In talking with nurses I have found that a certain percentage of consumptive patients are embittered by this prejudice against the disease, and being embittered, get reckless as to whether they scatter the disease or not. Their attitude is due to the lack of sympathy shown by persons associated with them. If these patients are approached by kind, sympathetic friends, and are both instructed and sympathised with, they may be made a most efficient aid in preventing the spread of tuberculosis."

(*To be continued.*)

